# A Woman with Three Breasts

**Published:** 1874-03

**Authors:** A. R. Kilpatrick

**Affiliations:** Texas


### A WOMAN WITH THREE BREASTS.

BY A. R. KILPATRICK, M.D., OF TEXAS.

I use the plain English instead of the Latin, as it sounds more natural to the ear.

I knew an elderly lady, living in Rapides Parish, La., named LACroix, who had three breasts. She was about sixty years old then, the mother of several children, all grown, and the males were all tall, large men, showing that they had been well nourished.

The breasts were in a line across the thorax; the two outside ones were in the normal positions usually occupied, while the third one was between them, directly over the sternum, and not quite as large as the others; but, she said there was no difference between it and the others, becoming enlarged during pregnancy, and in time of lactation, milk was secreted in it, and the children nursed from it.

Cases of this kind are reported by a few authors, and some were of women who have four mamma.

In P. Cazeaux's voluminous work on midwifery, the following brief notice of such cases is inserted by Prcf. S. Tarnier, who published the work with addenda, after the death of the illustrious author.

"The left breast is often larger than the right one. Curious anomalies, also, sometimes come under observation. Thus, women are reported having four breasts, and I have myself met with an instance of this kind in a woman who died at the Maternity Hospital. Two breasts of the usual size occupied their normal position, whilst two others, as fully developed, were situated on the upper and lateral parts of the abdomen, on the same vertical line with the thoracic ones. At the autopsy, I found abundance of glanlular tissue in all four of the breasts, which also contained milk."

